# Surgical Site Infections After External Ventricular Drain Placement: Incidence, Patient-Related, and Periprocedural Risk Factors

**DOI:** 10.1227/neuprac.0000000000000264

**Published:** 2026-09-02

**Authors:** Aron Alakmeh, Daniel de Wilde, Vittorio Stumpo, Stefanos Voglis, Flavio Vasella, Giovanna Brandi, Luca Regli, Carlo Serra, Victor E. Staartjes

**Affiliations:** 1 Machine Intelligence in Clinical Neuroscience and Microsurgical Neuroanatomy (MICN) Laboratory, Department of Neurosurgery, Clinical Neuroscience Center, University Hospital Zurich, University of Zurich, Zurich, Switzerland;; 2 Institute for Intensive Care, University Hospital Zurich, University of Zurich, Zurich, Switzerland;; 3 Department of Clinical Neuroscience, Karolinska Institutet, Stockholm, Sweden;; 4 Capio Spine Center Stockholm, Löwenströmska Hospital, Upplands Väsby, Sweden

**Keywords:** Cerebrospinal fluid, External ventricular drain, Infection, Risk factors, Ventriculostomy, Incidence

## Abstract

**BACKGROUND AND OBJECTIVES:**

External ventricular drain (EVD) placement is a routine neurosurgical procedure for cerebrospinal fluid (CSF) diversion. Despite standardized procedures, a considerable risk of infection remains. While CSF EVD-related infections (EVD-RI) are common and have been extensively studied, surgical site infections (SSI) as a distinct subset are, at least when compared with CSF EVD-RI, relatively rare and remain poorly understood in the context of EVD placement. Hence, the aim of this study was to determine the incidence and risk factors of SSI after EVD placement.

**METHODS:**

All consecutive adult patients undergoing EVD placement at a single neurosurgical center between January 2014 and April 2022 were included in this study. SSIs were defined according to the Centers for Disease Control and Prevention criteria for SSIs and were subsequently classified and managed in accordance with the institutional neurosurgical SSI guidelines. Patient-related and procedure-related factors were assessed for their role as independent predictors or confounders using a purposeful variable selection procedure, as proposed by Hosmer and Lemeshow.

**RESULTS:**

A total of 384 patients (418 EVDs; 34 bilateral) were included in this study. The incidence of SSI was 2.9% per patient and 2.6% per catheter. Factors associated with a significantly higher risk of SSI after EVD placement included concurrent CSF EVD-RI (odds ratio [OR] = 40.50, 95% CI: 7.17-399.29; *P* < .001), obesity (OR = 21.40, 95% CI: 2.08-200.81, *P* = .006) and arteriovenous malformation (OR = 50.27, 95% CI: 3.06-1124.25, *P* = .007) as an indication for EVD placement, whereas angiography was associated with a significantly lower risk (OR = 0.073, 95% CI: 0.010-0.45, *P* = .005).

**CONCLUSION:**

Postoperative SSI after EVD placement is a relatively rare, potentially preventable, and clinically and economically relevant complication. Both patient- and procedure-related factors influence SSI risk, with obesity and concurrent CSF EVD-RI emerging as strong predictors, highlighting the importance of meticulous perioperative management and vigilant postoperative surveillance, particularly in these risk groups.

**ABBREVIATIONS:**
CCI: Charlson Comorbidity Index
EVD: external ventricular drain
EVD-RI: EVD-related infections
SSI: surgical site infections.

External ventricular drain (EVD) placement is among the most frequently performed procedures in neurosurgery and serves as an essential intervention for therapeutic cerebrospinal fluid (CSF) diversion. Common indications include traumatic brain injury, acute hydrocephalus, intraventricular hemorrhage, other conditions requiring temporary CSF drainage, and the need for intracranial pressure monitoring.^1^ Despite its widespread use, EVD placement carries a considerable risk of healthcare-associated infections, commonly referred to as ventriculostomy-related infections or EVD-related infections (EVD-RI).^2,3^ Of these, the vast majority are CSF infections. Reported CSF EVD-RI rates vary between 5% and 30% and are consistently associated with increased morbidity, prolonged hospitalization, and higher rates of reoperation and mortality.^4-14^

Although CSF EVD-RIs are common and have been extensively studied, a rare subset of EVD-RIs involving incisional/surgical site infections (SSI) has received comparatively little attention. SSIs typically begin with microbial contamination of the incision and may represent an early or localized form of EVD-RIs.^15^ Their clinical presentation ranges from superficial wound dehiscence to osteomyelitis, epidural, subdural, and parenchymal infections, which can present as severe, potentially life-threatening infections.^16^ SSIs have been associated with longer hospital stays, increased treatment costs, and higher mortality.^17^

Although SSIs are reported in approximately 1% to 4.5% of neurosurgical procedures and around 3% of CSF diversion surgeries, their incidence and underlying risk factors after EVD placement remain poorly understood.^16,18,19^ If risk factors of this relatively rare complication (rare due to the small incision, but infection made more likely by the often severely affected health state of patients requiring EVD insertion) are known, some SSIs in patients requiring EVD insertion could be prevented in at-risk subgroups. This study addresses this gap by determining the incidence of SSIs and identifying perioperative predictors within a large neurosurgical cohort.

## METHODS

### Study Design and Patient Selection

This single-center retrospective cohort study was conducted at Department of Neurosurgery, University Hospital Zurich. Ethical approval for the institutional research registry (KEK-ZH-PB-2017-00093) had been obtained previously, and all data used for the present analysis were sourced from this registry. The requirement for individual informed consent was waived.

### Patient Population

The study included all consecutive adult patients (≥18 years) who underwent placement of EVD at our institution between January 2014 and April 2022. Patients with incomplete data or missing postoperative computed tomography imaging were excluded.

### Data Collection

Data were extracted from electronic medical records using a standardized case report form. Collected variables encompassed patient-related factors such as demographic information (age, sex), Glasgow Coma Scale (GCS) at admission, Charlson Comorbidity Index (CCI) at admission, immunosuppression at admission, presence of intraventricular hemorrhage, and comorbidities such as dyslipidemia, diabetes mellitus, myocardial infarction, heart failure, peripheral vascular disease, stroke, pulmonary disease, liver disease, renal disease, rheumatic disease, solid tumors, metastatic tumors, obesity (body mass index ≥30), and smoking.

In addition, periprocedural factors including the indication for EVD placement, necessity for an angiography or an additional hemicraniectomy, administration of alteplase, the need for neuromonitoring, treatment modality for underlying pathology (surgical vs interventional procedure), administration of postoperative antibiotics due to another infection (non–EVD-RI), rebleeding within 24 hours, occurrence of early (≤7 days postoperatively) or late (>7 days postoperatively) EVD leak, EVD exchange, and time to EVD removal. In case of wound infection, the time from admission to infection, causative microorganisms, site of infection, type of positive bacterial confirmation, and need for revision surgery were also recorded.

CSF EVD-RI was defined as a suspected infection according to the Infectious Diseases Society of America criteria and the approach by Pietrzko et al,^20^ rather than being limited to microbiologically confirmed cases, as is often applied in the literature.^21,22^

### EVD Placement

EVD placement was performed by neurosurgeons under sterile conditions, either in the operating theater or within the neurocritical care unit. Using a right frontal linear or curvilinear incision, the catheter was advanced and subcutaneously tunneled for a distance exceeding 5 cm. Bactiseal® catheters, coated with 0.15% clindamycin and 0.054% rifampicin (Integra LifeSciences), were used as the standard catheter system. As perioperative prophylaxis, patients received a single dose of cefuroxime (1.5 or 3 g, depending on body weight). Routine CSF monitoring included sampling twice per week and additional sampling if EVD-RI was suspected. CSF collection was routinely performed directly from the EVD under sterile conditions.

### Definition of Wound Infection

Wound infection was defined according to the Centers for Disease Control and Prevention criteria for SSI, which includes clinical signs of local inflammation (erythema, swelling, or purulent discharge) and/or positive microbiological cultures from the surgical wound obtained within 30 days postoperatively.^23^ Only infections directly related to the burr hole surgical site were considered for analysis. SSIs were furthermore categorized into groups (1-4) according to the institutional neurosurgical postoperative wound infection guidelines: cutaneous and subcutaneous infections without (1b) or with (2) exposure of bone flap fragments or foreign material, epidural infections (3), or infections extending to the bone (osteomyelitis) (4). Naturally, wound dehiscence (1a) was not considered an SSI in this study. Management of the different SSI categories was performed in accordance with the institutional classification and typically included surgical wound revision, microbiological confirmation through wound swab in the presence of clinical signs of inflammation, and initiation of antibiotic therapy, depending on the SSI type.

### Statistical Analysis

Continuous variables were summarized as mean ± SD and categorical variables as counts and percentages. To identify independent predictors and potential confounders of SSI using binomial logistic regression models, a three-step purposeful variable selection procedure was applied as described by Hosmer et al and detailed by Bursac et al.^24,25^

In the first step, a univariable analysis was performed, and variables with *P* < .25 were included. In the second step, all variables identified in the univariable analysis were entered into a multivariable model. Variables with *P* < .1 were retained as independent predictors, whereas those with *P* < .25 were assessed for potential confounding effects. A variable was considered a confounder if its removal resulted in a >20% change in the coefficient of any remaining variable. In the third step, all variables identified as neither predictors nor confounders were reintroduced one by one and tested for their influence; those causing a >20% change in the coefficient of any remaining variable were also classified as confounders.

All identified variables in the purposeful variable selection procedure were included in the final logistic regression model, in which a two-tailed *P* of < .05 was seen as statistically significant. All statistical analyses and graph generation were conducted using R version 4.2.3 (R Foundation for Statistical Computing).

## RESULTS

### Patient Characteristics

Patient characteristics are presented in detail in Table 1. In total, 384 patients with 418 EVDs (34 patients with bilateral EVDs) were included in this study. A total of 53.6% (n = 206) of patients were female, and the mean age at admission was 60.2 (±14.0) years. Aneurysmal subarachnoid hemorrhage was the most common indication for EVD implantation, accounting for 61.7% (n = 238) of cases. At admission, the mean GCS score was 9.9 (±4.7), and the mean CCI was 2.6 (±2.4).

**TABLE 1.** Summary of Parameters Investigated for Significant Association With SSI Risk

Parameter	Total (n = 384)	No SSI (n = 373)	SSI (n = 11)
Patient female sex, n (%)	206 (53.6)	200 (53.6)	6 (54.5)
Patient age (y), mean (SD)	60.19 (14.0)	60.34 (13.9)	55.27 (16.16)
GCS at admission, mean (SD)	9.86 (4.7)	9.85 (4.8)	10.18 (4.5)
Immunosuppression at admission, n (%)	17 (4.4)	16 (4.3)	1 (9.1)
CCI at admission, mean (SD)	2.59 (2.4)	2.61 (2.4)	2.09 (1.97)
Comorbidities			
Hypertension, n (%)	166 (43.2)	161 (43.2)	5 (45.5)
Obesity, n (%)	16 (4.2)	14 (3.8)	2 (18.2)
Dyslipidemia, n (%)	42 (10.9)	41 (11.0)	1 (9.1)
Heart failure, n (%)	18 (4.7)	17 (4.6)	1 (9.1)
Myocardial infarction, n (%)	24 (6.2)	23 (6.2)	1 (9.1)
Peripheral vascular disease, n (%)	16 (4.2)	16 (4.3)	0 (0.0)
Stroke, n (%)	22 (5.7)	22 (5.9)	0 (0.0)
Lung disease, n (%)	37 (9.6)	36 (9.7)	1 (9.1)
Rheumatic disease, n (%)	17 (4.4)	16 (4.3)	1 (9.1)
Kidney disease, n (%)	9 (2.3)	9 (2.4)	0 (0.0)
Solid tumor, n (%)	23 (6.0)	22 (5.9)	1 (9.1)
Metastatic tumor, n (%)	15 (3.9)	15 (4.0)	0 (0.0)
Intraventricular hemorrhage, n (%)	300 (78.1)	294 (78.8)	6 (54.5)
Smoking, n (%)	75 (19.5)	71 (19.0)	4 (36.4)
Liver disease, n (%)			
Mild	7 (1.8)	7 (1.9)	0 (0.0)
Moderate or severe	9 (2.3)	9 (2.4)	0 (0.0)
Diabetes, n (%)			
Mild	25 (6.5)	23 (6.2)	2 (18.2)
Moderate or severe	4 (1.0)	4 (1.1)	0 (0.0)
Indication for EVD, n (%)			
Intracerebral bleeding	87 (22.7)	85 (22.8)	2 (18.2)
Aneurysmal subarachnoid hemorrhage	237 (61.7)	233 (62.5)	4 (36.4)
Nonaneurysmal subarachnoid hemorrhage	26 (6.8)	25 (6.7)	1 (9.1)
Arteriovenous malformation	22 (5.7)	20 (5.4)	2 (18.2)
Arteriovenous fistula	11 (2.9)	10 (2.7)	1 (9.1)
Other	1 (0.3)	0 (0.0)	1 (9.1)
Treatment type for intracranial hemorrhage, n (%)			
No intervention	160 (41.7)	156 (41.8)	4 (36.4)
Additional interventional procedure	140 (36.5)	139 (37.3)	1 (9.1)
Additional surgical procedure	84 (21.9)	78 (20.9)	6 (54.5)
Hemicraniectomy, n (%)	38 (9.9)	36 (9.7)	2 (18.2)
Neuromonitoring, n (%)	62 (16.1)	61 (16.4)	1 (9.1)
Angiography, n (%)	249 (64.8)	245 (65.7)	4 (36.4)
Administration of alteplase, n (%)	15 (3.9)	15 (4.0)	0 (0.0)
Postoperative antibiotic treatment, n (%)	269 (70.1)	262 (70.2)	7 (63.6)
Rebleeding, n (%)	23 (6.0)	23 (6.2)	0 (0.0)
Catheter indwelling time (d), mean (SD)	18.80 (15.6)	19.03 (15.69)	11.00 (7.31)
EVD leak (any timepoint), n (%)	63 (16.4)	58 (15.5)	5 (45.5)
Early EVD leak, n (%)	6 (1.6)	6 (1.6)	0 (0.0)
Late EVD leak, n (%)	57 (14.8)	52 (13.9)	5 (45.5)
Catheter exchange, n (%)	64 (16.7)	58 (15.5)	6 (54.5)
Concurrent EVD-related CSF infection, n (%)	101 (26.3)	92 (24.7)	9 (81.8)

CCI, Charlson Comorbidity Index; CSF, cerebrospinal fluid; EVD, external ventricular drain; GCS, Glasgow Coma Scale; SSI, surgical site infection.

Regarding the treatment of the underlying pathology, 21.9% (n = 84) of patients underwent an additional surgical procedure (aneurysm clipping, tumor extirpation), 36.5% (n = 140) underwent endovascular procedures (aneurysm coiling, dural fistula embolization), whereas in 41.7% (n = 160) of patients, no additional intervention was performed. A catheter leak was documented in 16.4% (n = 63), with the majority classified as late leaks (n = 57/63; 90.5%). 26.3% (n = 101) of patients developed an EVD-related CSF infection. The mean duration of catheterization was 18.8 (±15.6) days. A total of 70.1% (n = 269) of patients received postoperative antibiotic treatment for infections other than CSF EVD-RI.

### SSI Characteristics

Of the 384 patients included in the analysis, 373 (97.1%) did not develop an SSI, whereas 11 (2.9%) did. Considering that a total of 418 EVDs were implanted in 384 patients, this corresponds to 2.6% of all EVDs being associated with SSI.

Characteristics of SSIs in this study are shown in detail in Tables 2 and 3. In 10 of 11 cases (90.9%), a causative germ could be identified (in addition to clinical signs), whereas 1 of 11 (9.1%) was diagnosed only based on clear clinical signs of infection (purulent wound secretion). Among the detected pathogens, *Staphylococcus epidermidis* was most frequent (6/11, 54.5%), followed by *Escherichia coli* (3/11, 27.3%). Regarding diagnostic confirmation, 4 of 11 cases (36.4%) were positive by bacterial culture alone, whereas 6 of 11 cases (54.5%) showed positive results in both Gram stain and bacterial culture.

**TABLE 2.** Characteristics of SSI

Parameter	No. of SSI (total n = 11) (%)
Microbiological infection confirmation	10 (90.9)
Type of microbiological infection confirmation	
Gram stain and culture positive	6 (54.5)
Only culture positive	4 (36.4)
Site of wound infection	
Superficial/deep cutaneous	4 (36.4)
Subcutaneous	10 (90.9)
Bone	2 (18.2)
Epidural	1 (9.1)
Pathogen types	
Staphylococcus epidermidis	6 (54.5)
Escherichia coli	3 (27.3)
Klebsiella pneumoniae	1 (9.1)
Serratia marcescens	1 (9.1)
Cutibacterium acnes	1 (9.1)
Acinetobacter baumannii	1 (9.1)
Klebsiella oxytoca	1 (9.1)
Revision surgery	10 (90.9)

SSI, surgical site infection.

**TABLE 3.** Individual Patient, Periprocedural, and Infection Characteristics for the 11 SSI Cases

Patient no.	Patient and periprocedural characteristics												Infection characteristics					
	Age	Sex	Diabetes	Obesity^a^	CCI at admission	GCS at admission	Indication for EVD^a^	Treatment type	Angiography^a^	EVD leak	Concurrent CSF EVD-RI^a^	EVD indwelling time	Time to SSI	Test type	Germ type	Infection site	Wound classification	Revision surgery
1	54	W	No	No	2	3	aSAH	Interventional	Yes	No	No	23	21	Culture	*S. epidermidis*	Cut.-bone	4	Wound revision
2	74	W	Yes	Yes	6	8	ICB	None	No	Yes	Yes	6	6	Both	*K. pneumoniae*	Cut.-epidural	3	EVD exchange
3	42	M	No	No	0	15	aSAH	Surgical	No	No	Yes	15	15	None	—	Subcut.	1b	Wound revision
4	59	M	No	No	1	8	aSAH	Surgical	No	No	Yes	10	10	Culture	*S. epidermidis* and *E. coli*	Subcut.	1b	EVD exchange
5	24	W	No	No	0	10	AVF	None	No	Yes	Yes	18	19	Both	*S. epidermidis*	Subcut.	1b	Wound revision
6	73	W	Yes	No	4	9	ICB	Surgical	No	Yes	Yes	9	8	Both	*S. epidermidis* and *E. coli*	Subcut.	1b	EVD exchange
7	39	M	No	Yes	0	15	aSAH	Interventional	Yes	Yes	Yes	17	21	Both	*S. epidermidis* and *E. coli*	Subcut.	1b	Wound revision
8	65	M	No	No	2	12	AVM	None	Yes	No	Yes	4	4	Both	*S. marcescens*	Subcut.	1b	EVD exchange
9	75	W	No	No	4	14	naSAH	None	Yes	No	Yes	20	11	Culture	*S. epidermidis* and *C. acnes*	Subcut.	1b	Wound revision
10	51	M	No	No	3	15	Other	None	No	Yes	Yes	16	16	Both	*A. baumannii*	Cut.-subcut.	1b	Wound revision
11	52	M	No	No	1	3	AVM	Surgical	No	No	No	13	13	Culture	*K. oxytoca*	Cut.	1b	None

aSAH, aneurysmal subarachnoid hemorrhage; AVF, arteriovenous fistula; AVM, arteriovenous malformation; C., cutibacterium; CCI, Charlson Comorbidity Index; CSF, cerebrospinal fluid; Cut., cutaneous; EVD, external ventricular drain; EVD-RI, external ventricular drain–related infection; GCS, Glasgow Coma Scale; ICB, intracranial bleeding; naSAH, nonaneurysmal subarachnoid hemorrhage; SSI, surgical site infection; subcut., subcutaneous.

a Significant independent predictor in statistical analysis (P<.05).

The most frequent infection sites were subcutaneous tissue in 7 of 11 cases (63.6%) and cutaneous tissue in 4 of 11 (36.4%). Regarding surgical management, 6 of 11 patients (54.6%) underwent wound revision, 3 of 11 (27.3%) required an EVD exchange, and 1 of 11 (9.1%) was managed without revision surgery.

Most patients with SSI had a concomitant CSF EVD-RI (9/11, 81.8%). In most of these cases, the same pathogen was identified in both CSF and wound (SSI) cultures (7/9, 77.8%).

### Risk Factors of SSI

Based on purposeful variable selection, the following factors were identified as independent predictors of SSI: CSF EVD-RI, obesity, indication for EVD placement, and angiography. Specifically, concurrent CSF EVD-RI (odds ratio [OR] = 40.50, 95% CI: 7.17-399.29; *P* < .001), obesity (OR = 21.40, 95% CI: 2.08-200.81, *P* = .006), and arteriovenous malformation (AVM) (OR = 50.27, 95% CI: 3.06-1124.25, *P* = .007) as an indication for EVD placement were associated with a significantly higher risk, whereas angiography (OR = 0.073, 95% CI: 0.010-0.45, *P* = .005) was associated with a significantly lower risk of SSI. These results are summarized in Table 4.

**TABLE 4.** Independent Predictors Identified in the Purposeful Variable Selection Model

Parameter	Odds ratio	LCL	UCL	*P*-value
Concurrent CSF EVD-RI	40.50	7.17	399.29	<.001
Obesity	21.40	2.08	200.81	.006
Angiography	0.073	0.010	0.45	.005
Indication for EVD placement				
Intracerebral bleeding	Reference			
Aneurysmal subarachnoid hemorrhage	2.51	0.31	29.80	.41
Nonaneurysmal subarachnoid hemorrhage	6.58	0.21	152.79	.22
Arteriovenous malformation	50.27	3.06	1124.25	.007
Arteriovenous fistula	3.99	0.13	81.69	.36
Other	>1000	<0.001	∞	.99

CSF, cerebrospinal fluid; EVD, external ventricular drain; EVD-RI, external ventricular drain–related infection; LCL, lower confidence limit; UCL, upper confidence limit.

Data were right censored.

## DISCUSSION

In a large neurosurgical registry, patient-related and periprocedural risk factors were investigated for the development of SSIs in patients after EVD placement. This is the first study to apply a proper multivariable method (the purposeful variable selection method proposed by Hosmer and Lemeshow) with the aim of identifying independent risk factors of SSI.^24^

SSIs after cranial neurosurgical procedures are often regarded as indicators of surgical and institutional quality and are generally considered preventable.^16,26^ Nevertheless, even with standardized techniques and protocols, SSIs still occur, independent of surgeon experience and institutional quality standards. Therefore, this study focused on identifying patient-related and periprocedural factors contributing to SSI, specifically in the context of EVD placement, one of the most common neurosurgical procedures. The results indicate that comorbidity (obesity), the underlying pathology, and perioperative factors (including the need for angiography and postoperative development of a CSF EVD-RI) play key roles in the occurrence of SSIs among patients undergoing EVD placement.

### Independent Predictors and Clinical Implications

#### CSF Infections

Concurrent CSF EVD-RI was identified as the strongest predictor for SSI, suggesting a close interplay between deep ventricular and superficial wound infections. This is further supported by the observation that in most cases of concurrent CSF EVD-RI, the same pathogen was identified in both SSI and CSF cultures. However, although in most cases of SSI a concomitant CSF EVD-RI was present, the absolute incidence of SSI, even among patients with CSF EVD-RI, was low.

This discrepancy can be explained by the distinct immunological environments of the ventricular system and the skin or subcutaneous tissue at the incision site. CSF EVD-RIs primarily result from bacterial migration along the catheter tract from the skin inward or from intraluminal contamination during manipulations.^27^ They occur relatively frequently because of the limited immune defense of the CSF compartment and the high potential of the catheter surface for bacterial adhesion and biofilm formation.^27,28^ By contrast, the small scalp incision, located in a well-perfused area, and the fact that the surgery duration is relatively short, is unlikely to develop an SSI. This stands in contrast to other neurosurgical procedures, such as multilevel lumbar spine surgery, where larger incisions, deeper and less vascularized tissue, and proximity to contaminated areas predispose to SSI.^29-31^ In patients with CSF EVD-RI, however, bacterial spread along the catheter may secondarily seed the incision site, thereby enabling an infection in an otherwise low-risk wound. Taken together, these findings emphasize the importance of early detection and management of CSF EVD-RI to prevent secondary SSI.

Of note, the relatively high incidence of CSF EVD-RI in this cohort (falling at the upper end of rates reported in the literature) is likely attributable to the specific patient population, which predominantly comprised critically ill patients with subarachnoid or intracerebral hemorrhage requiring prolonged intensive care unit care and extended EVD utilization. In addition, CSF EVD-RI was defined based on suspected rather than exclusively microbiologically confirmed infection, as outlined in the Methods section.

#### Obesity

Obesity emerged as an independent predictor for increased SSI risk after EVD placement. This finding is consistent with previous literature demonstrating that elevated body mass index is associated with higher postoperative infection rates across various neurosurgical and non-neurosurgical procedures.^32-34^ Possible explanations include impaired wound healing because of reduced tissue perfusion, a higher prevalence of diabetes and metabolic dysregulation, and technical difficulties during surgery that may increase operative time and wound tension.^26^ From a clinical standpoint, this underlines the importance of meticulous perioperative hygiene, careful wound closure techniques, and heightened vigilance for early signs of infection in obese patients.

#### Angiography

Interestingly, angiography (eg, patients going into angiography/angioplasty/interventional spasmolysis for vasospasms) was associated with a significantly lower risk of SSI. Evidence regarding the relationship between angiography and postoperative infection risk in neurosurgical procedures remains scarce. For example, Stetler et al^35^ demonstrated that intraoperative angiography in patients with intracranial aneurysms did not increase the incidence of SSI. However, their study focused on patients undergoing operative aneurysm treatment rather than adjuvant angiography as in our study. A possible explanation for our finding could be that patients developing vasospasms and requiring angiography had a substantially higher risk of early mortality, thereby reducing the time window in which an SSI could manifest or be detected (competing risk bias).

#### Indication for EVD Placement

The indication for EVD placement was also identified as a significant determinant of SSI risk. Interestingly, patients with AVM exhibited markedly higher infection rates compared with those with other causes for intracerebral hemorrhage. This association may reflect longer and more complex surgical procedures in patients with AVM, leading to higher cumulative SSI risk.^30^ Often, when a craniotomy for supratentorial AVMs is performed, eg, a pterional skin incision also has to be connected to the EVD incision or at least the incision is placed close to the existing EVD incision. Combined with longer operating times in AVMs, this may indeed lead to a higher cumulative vulnerability. These results highlight the need for tailored infection prevention strategies in high-risk indications, such as strict adherence to sterile handling and shorter drainage durations whenever clinically feasible.

### SSI Characteristics

In this cohort, *S. epidermidis* represented the most common causative pathogen of SSI, followed by Gram-negative species such as *E. coli*. This distribution is in line with previous literature on SSI in other neurosurgical procedures and CSF EVD-RI, where coagulase-negative staphylococci have consistently been identified as the predominant organisms.^16,32,36^ The detection of Gram-negative bacteria, though less frequent, underscores the importance of broad initial empirical coverage in case of SSI, particularly in patients with prolonged hospitalization or intensive care exposure. Most infections involved the superficial subcutaneous layers, whereas deeper structures such as bone or epidural space were rarely affected, which parallels previous findings indicating that true deep wound infections are relatively uncommon in cranial procedures.^16^

### Limitations

As SSIs are generally rare in neurosurgical procedures, including EVD placement procedures as confirmed in this study, a relatively large data set was deemed necessary to enable meaningful analysis.^16^ We provide a relatively large and, more importantly, relatively granularly studied cohort of patients undergoing EVD placement. Nevertheless, an even larger cohort would have been desirable to further strengthen the analyses. Another limitation is the lack of a universally standardized definition of SSI in neurosurgical research. Variations in diagnostic thresholds and inclusion of microbiological vs purely clinical findings across studies may also limit comparability of infection rates.^23^ A further limitation of the study is that only patient-related and periprocedural factors were considered. Accordingly, potentially relevant variables such as procedure setting (operating room vs intensive care unit) and operative duration were not included in the analysis.

## CONCLUSIONS

SSIs after EVD placement are relatively rare but represent clinically significant complications that may substantially affect patient outcomes. Both patient-related factors, particularly obesity, and periprocedural factors such as concomitant CSF EVD-RI strongly influence the risk of SSI. These findings highlight the importance of strict adherence to sterility in EVD placement and management, early recognition and treatment of CSF EVD-RI, and tailored preventive measures in obese patients.

## Data Availability

The data in support of our findings can be obtained upon reasonable request from the corresponding author.
